# Supplementary material for: Patterns of Intron Gain and Loss in Fungi
Source: PLoS Biol. 2004 Nov 30;2(12):e422. doi: 10.1371/journal.pbio.0020422 (PMC532390; doi:10.1371/journal.pbio.0020422)
Supplement: Table S1 — Also available at http://genes.mit.edu/NielsenEtAl/. (4.3 MB ZIP). [file pbio.0020422.st001.zip › NielsenEtAl/html/1179.html]

AN2526.1.NCU03608.1.MG01808.1.FG10118.1


```
 CLUSTAL W (1.82) Multiple Sequence Alignments - Introns Inserted


Sequence 1: NCU03608.1	402 aa
Sequence 2: MG01808.1	400 aa
Sequence 3: FG10118.1	405 aa
Sequence 4: AN2526.1	400 aa
Alignment Length: 405 aa
Number Identitical Residues: 294 aa
Alignment Score (without introns) 12532


MG01808.1 	MSARGFSKALRPMARQLAT-PAVQRRTFVAAS-SMVRATR--KAAVA-PTQQQIRGVKTM
NCU03608.1	MAARNCTKALRPLARQLAT-PAVQRRTFVAAA-SAVRASVAVKAVAA-PARQQVRGVKTM
FG10118.1 	MASRNLSKALRGPARQLASAPRVQQRTFVSAARAAVRAGAVARPVASGPIQQQIRGVKTM
AN2526.1  	MASRGLPRALR-VARVAAP-RSVVSAALPRPSLAKIAANAVPRVAAP---VVPVRGVKTI
          	*::*. .:***  **  *.   *   ::  .: : : *    : ...      :*****:

MG01808.1 	DFAGHKEQVWE1RADWPKEKLL0EYFKDDTLALIGYGSQGHGQGLNLRDNGLNVIIGVRK
NCU03608.1	DFAGHKEEVHE1RADWPAEKLL0DYFKNDTLALIGYGSQGHGQGLNLRDNGLNVIVGVRK
FG10118.1 	DFAGHKEDVYE1RADWPQEKLL~EYFKNDTLALIGYGSQGHGQGLNLRDNGLNVIVGVRK
AN2526.1  	SFADSQETVYE~RADWPREKLQ~EYFKNDTLALIGYGSQGHGQGLNLRDQGLNVIVGVRK
          	.**. :* * * ***** ***  :***:*********************:*****:****

MG01808.1 	DGKSWKDAVQDGWVPGKNLFEVDEAISRGTVIMNLLSDAAQSETWPALKPQITKGK0TLY
NCU03608.1	NGKSWEDAIQDGWVPGKNLFDVDEAISRGTIVMNLLSDAAQSETWPHIKPQITKGK0TLY
FG10118.1 	NGKSWKDAEQDGWVAGKNLFDVDEAIGRGTIVMNLLSDAAQSETWPAIKPQLVKGK0TLY
AN2526.1  	DGASWKEAIQDGWVPGKNLFEVTEAVQKGTIVMNLLSDAAQSETWPTLKPLITKGK~TLY
          	:* **::* *****.*****:* **: :**::************** :** :.*** ***

MG01808.1 	FSHGFSPVFKDLTKVEVPTDVDVILCAPKGSGRTVRSLFREGRGINSSFAVYQDVTGEAE
NCU03608.1	FSHGFSPVFKDLTKVEVPTDVDVILVAPKGSGRTVRSLFREGRGINSSFAVYQDVTGKAK
FG10118.1 	FSHGFSPVFKDLTKVEVPTDIDVILCAPKGSGRTVRSLFREGRGINSSFAVYQDVTGKAE
AN2526.1  	FSHGFSPVFKEKTKVDVPSDVDVILVAPKGSGRTVRTLFREGRGINSSIAVFQDVTGNAK
          	**********: ***:**:*:**** **********:***********:**:*****:*:

MG01808.1 	EKAIALGVAIGSGYLYKTTFEKEVYSDLYGERGCLMGGIHGMFLAQYEVLRERGHSPSEA
NCU03608.1	EKAVALGVAVGSGYLYETTFEKEVYSDLYGERGCLMGGIHGMFLAQYEVLRERGHSPSEA
FG10118.1 	EKAIALGVAIGSGYLYKTTFEKEVYSDLYGERGCLMGGIHGMFLAQYEVLRERGHSPSEA
AN2526.1  	EKAIAMGVAVGSGYLYETTFEKEVYSDLYGERGCLMGGIHGMFLAQYEVLRERGHSPSEA
          	***:*:***:******:*******************************************

MG01808.1 	FNETVEEATQSLYPLIGANGMDWMYEACSTTARRGAIDWSPRFKDALKPVFNQLYDSVKD
NCU03608.1	FNETVEEATQSLYPLIGAHGMDWMFDACSTTARRGAIDWTPKFKDALKPVFNNLYDSVKN
FG10118.1 	FNETVEEATQSLYPLIGANGMDWMYEACSTTARRGAIDWSPKFKDALKPVFNSLYDAVKD
AN2526.1  	FNETVEEATQSLYPLIGANGMDWMYAACSTTARRGAIDWSSRFKDNLKPLFNELYDSVRD
          	******************:*****: *************:.:*** ***:**.***:*::

MG01808.1 	GSETQRSLDYNSQPDYREKYEAEMEEIRNLEIWRAGKAVR~SLRPENQK
NCU03608.1	GDETKRSLEYNSQPDYRERYEAELDEIRNLEIWRAGKAVR2SLRPENQK
FG10118.1 	GSETKRSLEYNSQPDYREKYEAEMKEIRELEIWRAGKAVR~SLRPENQK
AN2526.1  	GTETQRSLDYNSQKDYREKYEKEMQEIRDLEIWRAGKAVR~SLRPENQK
          	* **:***:**** ****:** *:.***:*********** ********
```
